# Supplementary material for: Frequency of ventricular arrhythmias in apparently healthy, large breed dogs during seven-day Holter monitoring
Source: PLoS One. 2025 Jun 2;20(5):e0319886. doi: 10.1371/journal.pone.0319886 (PMC12129169; doi:10.1371/journal.pone.0319886)
Supplement: S1 Table — (PDF) [file pone.0319886.s001.pdf]

S Table 1: The frequency of ventricular arrhythmias and their complexities during 7-day Holter monitoring in healthy, large breed dogs.

Dog number	Maximum number of VPCs	Minimum number VPCs	Range of Couplets	Range of triplets	Range of VT	Max Ventricular escapes	Minimum number of pauses	Maximum number of pauses	R on T VPCs
1	0	0	0	0	0	0	7	86	0
2	0	0	0	0	0	0	3	240	0
3	4	0	0-1	0	0-1	0	174	5461	0
4	2	1	0-1	0	0	2	78	439	0
5	2	1	0	0	0	0	31	399	0
6	1	0	0-1	0	0	1	80	483	0
7	1	0	0-1	0	0-2	1	217	2119	0
8	15	0	0	0	0	1	108	595	0
9	37	5	0-1	0-1	0	1	0	3	0
10	1	0	0-1	0	0	0	976	2623	0
11	13	4	0-1	0	0	1	162	1839	0
12	2	0	0	0	0	0	6	102	0
13	61	4	0-3	0	0	1	72	737	0
14	0	0	0	0	0	0	16	2335	0
15	1	0	0-1	0	0	0	80	515	0
16	7	0	0	0	0	133	0	307	0
17	2	0	0-1	0	0	2	950	8279	0
18	2	0	0	0	0	0	0	96	0
19	3	0	0-1	0-1	0	3	29	1043	0
20	3	0	0-1	0-1	0	2	4	313	0
21	51	25	0-1	0-1	0	1	12	499	0
22	2	0	0-1	0	0	0	574	2258	0
23	39	3	0-1	0	0-1	2	20	358	0
24	2	0	0-12	0-1	0	129	114	1087	0
25	1	0	0	0	0	2	70	385	0
26	1	0	0	0	0	1	24	137	0
27	1	0	0-1	0	0	1	43	1694	0
28	1	0	0	0	0	0	153	1017	0
29	6	0	0	0	0	1	977	2669	0
