# Supplementary material for: Frequency of ventricular arrhythmias in apparently healthy, large breed dogs during seven-day Holter monitoring
Source: PLoS One. 2025 Jun 2;20(5):e0319886. doi: 10.1371/journal.pone.0319886 (PMC12129169; doi:10.1371/journal.pone.0319886)
Supplement: Attachment — Submitted filename: Frequency of ventricular arrhythmias in apparently healthy.docx [file pone.0319886.s002.docx]

Frequency of ventricular arrhythmias in apparently healthy, large breed dogs during 7 day Holter monitoring

Introduction

Line 24 – The variability was taken from just 4 dogs. I understand the point you’re trying to make here but we also have to be careful not to over-emphasise a single result as this could mislead the casual reader. I would be happier if you changed it to: significant day to day variation (50-93%) in 4 dogs with over twenty…

Line 26-27 – I think your statement about age and freq of vent arrhythmias is quite bold given that the study population was predominately older dogs. Perhaps soften this to: No corelation was found between age and VPC frequency (P = 0.409) in this population of predominantly older dogs.

Line 40 – ref 4 – would be ok if placed next to ref 1 but this study did not investigate anti-arrhythmic drug therapy

Line 41 – gold standard screening would also involve echocardiography so consider changing to: ambulatory ECG is one component of gold standard screening to identify…

- Line 46 – consider adding refs for Dobermanns, Great Danes. Maybe these ones: Currently ref 18 - Gunasekaran, T et al. “Comparison of single- versus seven-day Holter analysis for the identification of dilated cardiomyopathy predictive criteria in apparently healthy Doberman Pinscher dogs.” *Journal of veterinary cardiology : the official journal of the European Society of Veterinary Cardiology* vol. 27 (2020): 78-87. doi:10.1016/j.jvc.2020.01.003
- El Sharkawy, S et al. “Long-term outcome and troponin I concentrations in Great Danes screened for dilated cardiomyopathy: an observational retrospective epidemiological study.” *Journal of veterinary cardiology : the official journal of the European Society of Veterinary Cardiology* vol. 47 (2023): 1-13. doi:10.1016/j.jvc.2023.03.003

Line 50 – ref 14 – not in English. Perhaps a bit harsh and you may be fluent in German but if you haven’t read the full text can you justify use as a reference?

Line 67 – ref 4 does look at a range of dog breeds although I appreciate your study is prospective

Line 103 / table 1 - Why did you classify idioventricular rhythm as ≥4 beats at ≤50bpm and, more controversially, VT as ≥4 beats at ≥50bpm. If you stick with idioventricular rhythm at <50bpm, do you then add accelerated idioventricular rhythm for 50-180bpm and VT for >180bpm?

Lines 110-113 – how did you classify trigeminy / bigeminy.

Line 137 – the predominance of older dogs in this study is a possible limitation

Lines 138-140 – the total number of dogs here is 29 not 31. I know 2 dogs were censored but this is not clear until lines 142-152. Consider re-writing so the numbers work.

Line 155 – table title – considering stating that these data are from 29 apparently healthy large breed…

Line 156 – Table 2 – I would consider moving the (seven days) to above the right hand collum containing the numbers.

Would you consider the dog with 399 ventricular escape beats to be normal? On reflection could this dog have sinus node dysfunction?

Line 167 – I’m not sure how meaningful this is when taken from only 4 dogs.

Lines 173-181 – were these dogs with complex ectopy normal?

Line 179 – see previous comment about rate of VT – for me 144bpm would be an accelerated idioventricular rhythm.

Line 181 and also 213 – the way the text reads to me is quite dismissive about the effects of age but, in my opinion, this relatively small study with predominantly older dogs is not best placed to fully evaluate the effect of age on the freq of ventricular arrhythmias so perhaps consider phrasing a bit more cautiously. Consider adding this ref: Cruz Aleixo, Amanda Sarita et al. “Scaling Relationships Among Heart Rate, Electrocardiography Parameters, and Body Weight.” *Topics in companion animal medicine*vol. 32,2 (2017): 66-71. doi:10.1053/j.tcam.2017.06.002

Lines 194 - 196 – Compare this with the results described in Ref 4

Line 219 – I would also consider the dogs with complex ventricular ectopy to be abnormal.
